# Management of degenerative rotator cuff tears: a review and treatment strategy

**DOI:** 10.1186/1758-2555-4-48

**Published:** 2012-12-14

**Authors:** Nicholas D Clement, Yuan X Nie, Julie M McBirnie

**Affiliations:** 1 Royal Infirmary of Edinburgh, Little France, Edinburgh EH16 4SA, Scotland

## Abstract

The aim of this review was to present an over view of degenerative rotator cuff tears and a suggested management protocol based upon current evidence. Degenerative rotator cuff tears are common and are a major cause of pain and shoulder dysfunction. The management of these tears is controversial, as to whether they should be managed non-operatively or operatively. In addition when operative intervention is undertaken, there is question as to what technique of repair should be used. This review describes the epidemiology and natural history of degenerative rotator cuff tears. The management options, and the evidence to support these, are reviewed. We also present our preferred management protocol and method, if applicable, for surgical fixation of degenerative rotator cuff tears.

## Introduction

The earliest published description of a rotator cuff tear was by Alexander Munro some 220 years ago in 1788, describing a “*hole with ragged edges in the capsular ligament of the humerus*” [1]. Since this description there has been little agreement amongst orthopaedic surgeons regarding the exact indications for surgical repair of a torn degenerative rotator cuff [2]. The purpose of this review was to present an overview of degenerative rotator cuff tears and a suggested management protocol based upon current evidence.

## Epidemiology

The prevalence of rotator cuff disease increases with age, with 4% of asymptomatic patients aged less than 40 years and 54% of patients aged 60 years or over, having partial or complete tears of the rotator cuff on magnetic resonance scanning [3]. Ultrasound scanning has demonstrated that 13% of the population in the fifth decade, 20% in the sixth decade and 31% in the seventh decade of life have a rotator cuff tear [4]. Yamaguchi et al. [5] demonstrated that more than half of asymptomatic rotator cuff tears become symptomatic within 3 years and progressed in size during this time period.

## Evaluation and diagnosis

Degenerative rotator cuff tears tend to occur in older patients (>50 years old) and often have no history of trauma, presenting with progressive shoulder pain and/or dysfunction [6]. Examination may reveal atrophy around the shoulder girdle secondary to chronic disuse, typically in the supraspinatus and infraspinatus fosse [6]. Range of movement should be assessed, where active movement may be limited but generally passive is full [6]. Neers sign and Hawkins signs can be used to assess for impingement of the rotator cuff [7]. More specifically horn blowers sign, Jobe’s and Gerber’s belly press tests assess specific rotator cuff muscles; teres minor, supraspinatous, and subscapularis respectively [8]. Multiple imaging modalities are available to assess the status of the rotator cuff. Plain radiographs enable assessment of the acromiohumeral space (normally 7 to 14mm), acromial morphology, and the glenohumeral joint, which can be used to grade the rotator cuff arthropathy [9]. Ultrasound allows dynamic assessment of the rotator cuff with no radiation exposure, however magnetic resonance imaging (MRI) remains the gold standard in the radiographic assessment of the rotator cuff [10].

## The natural history of a rotator cuff tear

**Figure 1:**
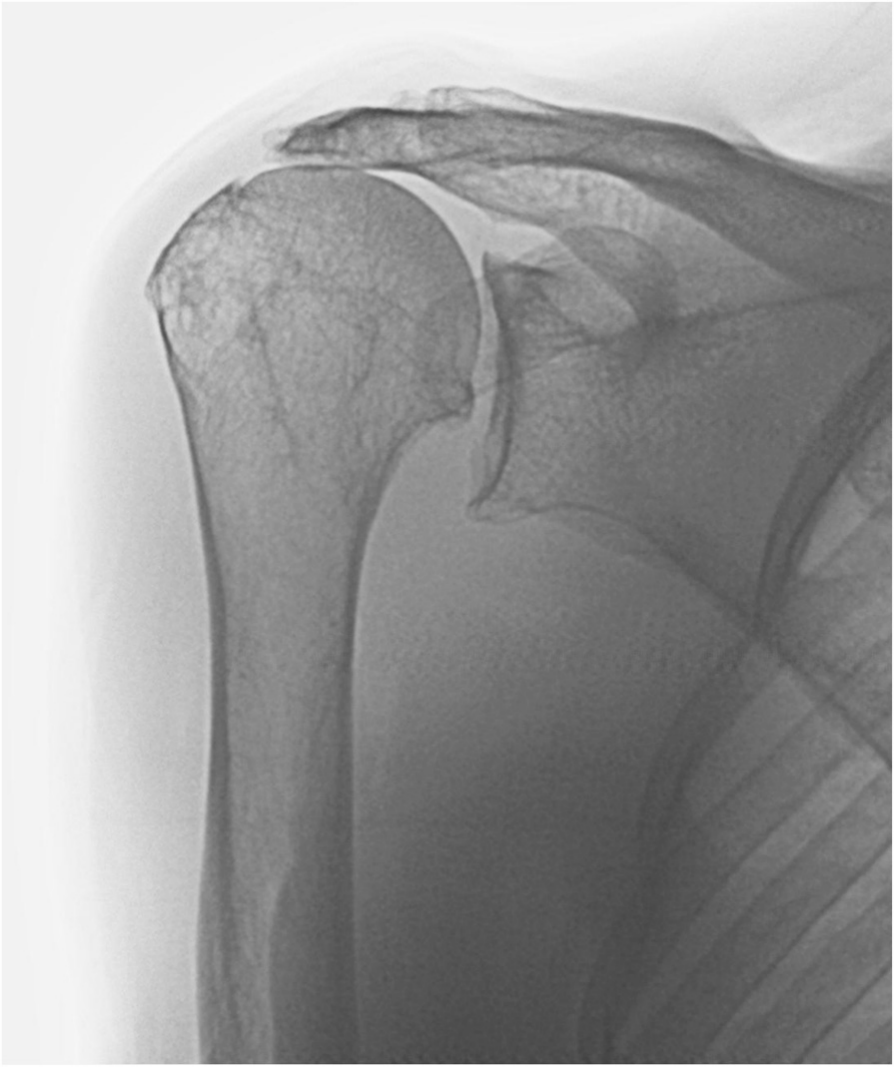
**Rotator cuff arthropathy secondary to rotator cuff disease, demonstrating acetabulisation, with a concave deformity of the acromion, and narrowing of the acromiohumeral distance to less than 6mm (Hamada’s grade 3 [**[9]**]).**

Neer originally described three stages of rotator cuff disease [11]. Stage I occurring in patients younger than 25 years with oedema and hemorrhage of the tendon and bursa. Stage II involves tendinitis and fibrosis of the rotator cuff in patients 25 to 40 years of age. Stage III involves tearing of the rotator cuff, either partial or full-thickness, and occurs in patients older than 40 years of age. Whether the pathological changes observed in the rotator cuff is secondary to intrinsic tendon degeneration and/or extrinsic mechanical impingement is a matter of debate. Yamanaka et al. [12] demonstrated that 10% of partial-thickness tears heal and 10% become smaller, but 53% of tears will propagate and 28% progress to full-thickness tears. Full-thickness rotator cuff tears do not heal spontaneously, and may progress with time [5,13,14]. Basic research has demonstrated that the number of procollagen alpha 1 positive tendon cells in the edge of the tear decrease markedly 4 months after the tear [15], and hence may explain the failure to heal and progression in some patients. This is thought to be due to poor vascularization within the degenerate rotator cuff as well as the intra-articular environment which can inhibit healing [16-18]. A number of patients will develop an irreparable rotator cuff tear due to progression of the tear and tendon retraction, and some patients will go on to develop secondary degenerative changes of the glenohumeral joint termed rotator cuff arthropathy (Figure 1) [19]. Early repair of the rotator cuff tear may prevent progression of the tear and avoid cuff arthropathy which is difficult to manage [20]. Even when the rotator cuff tear has progressed to a massive tear repair has been shown to avert radiographic deterioration and resultant cuff tear arthropathy [21].

## Management

The management of a rotator cuff tear is multifaceted. Conservative management includes analgesia and anti-inflammatory medications, physical therapy, activity modification and subacromial injections of local anaesthetic and/or steroid. Injection of hyaluronate is advocated by some authors for complete rotator cuff tears, but a randomized control trial found it to be no more effective than a steroid injection [22]. More recently however Chou et al. demonstrated a significant improvement in shoulder function at 6 weeks following injection with hyaluronate compare with placebo for partial tears [23]. Operative interventions include arthroscopic debridement of the tear or repair of the torn rotator cuff, with or without subacromial decompression. Most reports in the literature are procedure oriented, consisting of retrospective single surgeon series with limited numbers of patients. A Cochrane review performed in 2004 analysed interventions for rotator cuff tears and concluded that there is little evidence to support or refute the efficacy of commonly used treatment methods [24].

## A suggested approach to management of a rotator cuff tear

The aim in managing a rotator cuff tear is to reduce pain and improve function. The evidence for conservative management of a rotator cuff tear dictates an initial period, of at least 6 weeks to 3 months, of non-operative treatment unless there is evidence of an acute tear in a younger patient [25-27]. Prolonged conservative management in symptomatic patients can have negative consequences. These include increase in tear size, tear retraction, increased difficulty of repair [28,29] and muscle atrophy with fatty infiltration, all of which can result in a diminished outcome [29-32].

Despite limited evidence, physiotherapy is the mainstay of conservative management of rotator cuff tears. An ultrasound or MRI scan may be obtained for patients with persistent symptoms that have not improved after 2 to 3 months of conservative management. There is no good evidence for or against steroid injection in the management of rotator cuff tears, although empirically these do seem to have a positive effect in some patients. Multiple injections should be avoided however, especially if there is a diagnosed rotator cuff tear that is potentially repairable.

Initial radiographic assessment includes an anteroposterior, scapulolateral, and axillary view. If a rotator cuff tear is suspected based on clinical assessment, an ultrasound or MRI scan can be obtained. An ultrasound scan offers dynamic assessment of the rotator cuff with less expense, relative to a MRI scan, but it is operator dependent. A MRI scan can also evaluate tear size and retraction, but in addition the rotator cuff muscles can be assessed for fatty atrophy which predicts outcome after repair.

## Conservative management

Symptomatic rotator cuff tears treated conservatively can give a baseline to which the outcome after surgical intervention can be compared. Bartolozzi et al. [25] in a study of 136 patients managed conservatively with symptomatic rotator cuff disease identified that full-thickness tears greater than 1cm^2^, symptoms persisting more than 1 year, and functional impairment and weakness were associated with a worse outcome. They recommended that surgery be considered in these patients with those risk factors. In contrast however, they found no association between age and functional outcome [25]. Itoi and Tabata [27] reported 62 cuff tears in 54 patients that were treated conservatively and found that 72% of patients had good or excellent results at an average of 3.4 years. This however was a selected cohort of patients presenting with mild pain and minimal functional deficit. Bokor et al. [26] reported that 74% of patients with confirmed rotator cuff tears managed conservatively had minimal or no pain at 7 years and 86% were satisfied with their result. In this study, patients who failed conservative treatment and went on to have surgery were excluded, which introduces an obvious selection bias. Samilson and Binder [33] report the largest series of conservatively managed full-thickness rotator cuff tears (n=292), demonstrating that 72% of shoulders had more than 150º of abduction after treatment but 40% were rated as having a fair or poor outcome. Hawkins and Dunlop [34] reported a smaller series of 33 patients managed conservatively. No patients were excluded and unsatisfactory results occurred in 14 of 33 (42%) with 12 patients eventually undergoing surgery. Patients with an insurance claim were less likely to be satisfied.

## Operative management

Repair of a torn rotator cuff has been shown to give predictable pain relief and functional improvement, with good overall patient satisfaction [35]. The results of open, mini-open and arthroscopic rotator cuff repair have all generally been favourable, but approximately 38% of patients suffer a post-operative complication [36]. Re-rupture rates of 13% [37] to 68% [38] have been reported after rotator cuff repair, however patients suffering a re-rupture still have significant improvement in pain and function [39]. The re-rupture rate, as assessed by MRI is 20% to 39% [40-42] and in larger tears the rate at 2 years is nearly double this (41% to 94%) [43-45]. Patients with an intact repair have significantly better outcomes [41,44]. The outcome of revision surgery for symptomatic failed primary repairs is inferior to successful primary repair, with only 69% of patients being satisfied [46]. Despite the risk of complications and tendon re-rupture, rotator cuff repair predictably reduces pain and improves strength and function in symptomatic patients [47].

**Figure 2:**
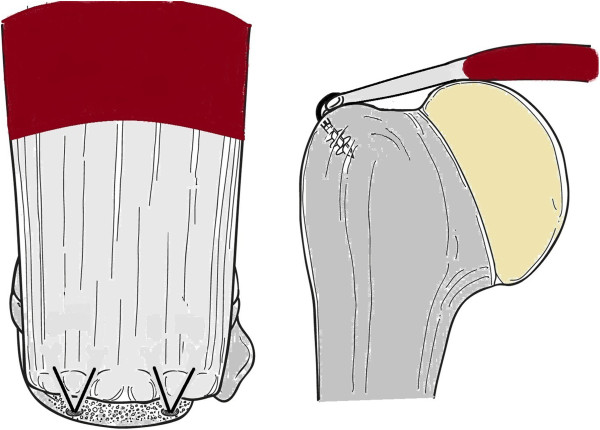
A single row repair on the lateral aspect of the foot print.

**Figure 3:**
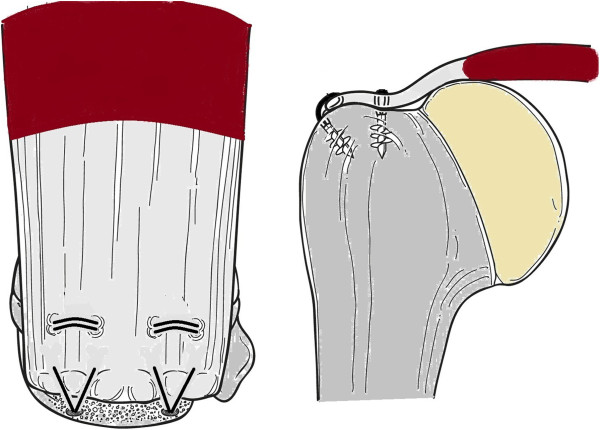
A double row repair securing the rotator cuff tendon into the foot print.

**Figure 4:**
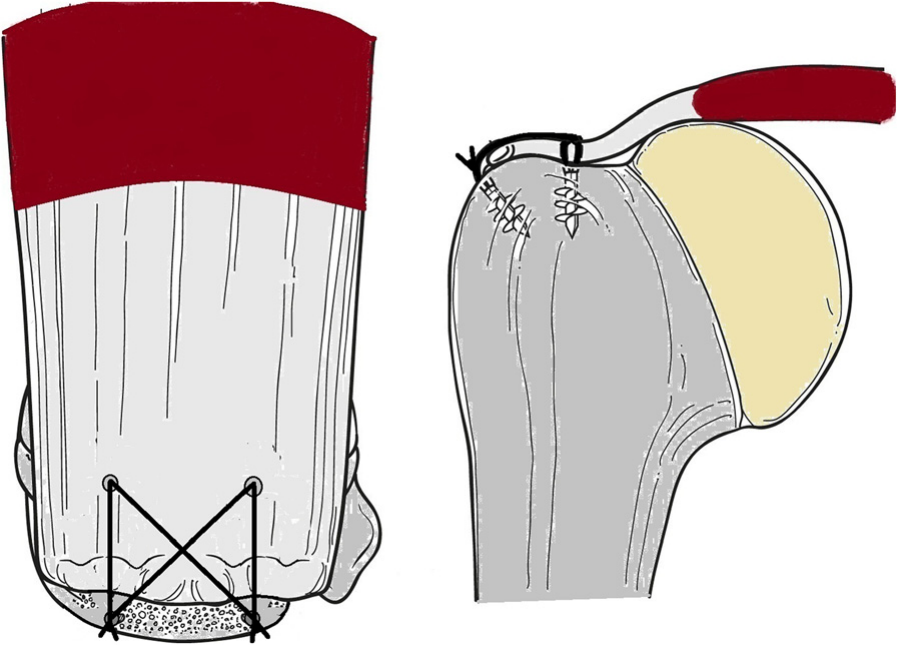
A double row bridging repair securing the rotator cuff tendon into the foot print.

There is great debate throughout the literature as to whether arthroscopic rotator cuff repair is superior to that performed through a mini-open approach. A recent systematic review by Lindley and Jones [48] found no statistically significant difference in postoperative outcome or incidence of re-ruptures of those rotator cuff tendons repaired arthroscopically versus using the mini-open repair technique. There was, however, decreased post-operative pain in the short-term for patients who underwent arthroscopic repair. In addition to the surgical approach for repair, the technique of cuff repair is also contested. Options for this include a single row (Figure 2), double row (Figure 3), or suture bridge repair (Figure 4). Trappey and Gartsman [49] performed a systematic review of the literature to answer this question. They identified four randomised control trials, all of which demonstrated no difference in the clinical outcome between single or double row repairs. The most recent study, by Gartsman et al. [50], demonstrated a significant difference in the re-rupture rate when comparing a single row repair (20% re-rupture) with a double row suture bridge technique (7% re-rupture) (Figure 4). They did not assess clinical outcome and this improved re-rupture rate may translate into a superior patient outcome. Trappey and Gartsman [49] suggest that more sophisticated outcome analyses may be needed to confirm the superiority of double row repairs.

## Conclusion

Most of the guiding principles used for decision-making in treating rotator cuff disease are based on limited evidence and minimal science. Factors that seem to be important include duration of symptoms, weakness, size of the tear, and muscle atrophy. If surgery is performed, either by a mini-open or arthroscopic technique, a double row bridging repair seems to be biomechanically stronger, provided this can be performed in a tension-free environment. At this point in time there is no functional evidence to support double row repair over single row repair, however the re-rupture rate is diminished after a double row repair.

## Competing interests

The authors declare that they have no competing interests.

## Authors’ contributions

NDC and YXN conducted the literature review, analysed the data, and composed the paper. JMM, as senior author and expert in shoulder arthroscopy, were involved in editing the final manuscript and given approval to the final version submitted for publication. All authors have read and approved the final manuscript.
